# Host-Parasite Interactions in Some Fish Species

**DOI:** 10.1155/2012/237280

**Published:** 2012-07-31

**Authors:** R. A. Khan

**Affiliations:** Department of Biology, Memorial University of Newfoundland, St. John's, NL, Canada A1B 3X9

## Abstract

Host-parasite interactions are complex, compounded by factors that are capable of shifting the balance in either direction. The host's age, behaviour, immunological status, and environmental change can affect the association that is beneficial to the host whereas evasion of the host's immune response favours the parasite. In fish, some infections that induce mortality are age and temperature dependent. Environmental change, especially habitat degradation by anthropogenic pollutants and oceanographic alterations induced by climatic, can influence parasitic-host interaction. The outcome of these associations will hinge on susceptibility and resistance.

## 1. Introduction

 Interaction between hosts and parasites is a complex relationship that can favour one or the other depending on a number of factors. Initially, the parasite attempts to establish itself in the host while the latter resists the infection via its defense mechanisms. Consequently, host susceptibility and resistance will determine whether or not the infection becomes established. In some fish species, the host's age, behaviour, physiological and immunological condition, proximity to shore, location in the water column, and feeding habits could affect the relationship while the parasite's mode of entry, ability to evade its host's defense, nutritional requirements, and living in a site where the immune response is reduced and mimicking its host's protein composition are factors that influence susceptibility and infectivity. There are also environmental variables such as water temperature, crowding, and habitat changes that could affect the interaction. Moreover, this interrelationship between hosts and parasites has evolved some associations resulting in host specificity, latitudinal gradients, and diversity in communities and siblings within one species [1–4]. Other associations can exist in a harmonious compromise whereas host avoidance of the parasite to prevent an infection or evasion by the parasite of the host's immune system can occur. For example, certain song birds in Hawaii avoid *Plasmodium* sp. infections from ground-feeding mosquitoes by remaining high up in the forest canopy [5]. In contrast, an African trypanosome, *Trypanosoma brucei* complex, sheds its surface coats in response to its host's antibodies [6]. Some parasites have evolved methods, such as circadian rhythms, to maximize transmission to uninfected hosts. Synchrony of peak abundance of microfilaria of *Wucheraria bancrofti* and mature gamonts of *Plasmodium* species in the peripheral blood of humans, when female mosquitoes are actively seeking a blood meal at night, increases infectivity and development in the vector [7]. McCarthy [8] also noted that a hemoflagellate, *T. murmanensis*, was more abundant in blood from the gills of Atlantic cod, *Gadus morhua*, at night when the vector, a marine leech, *Johanssonia arctica*, fed on its piscine hosts than during daylight. The leech, a deep-water (>90 meters) species inhabiting the benthic zone where light is probably negligible, was significantly more abundant than at shallow depths (30 meters) where some light penetrates [9]. Some parasites can also avoid their host's defence mechanisms. Cysticerci of *Taenia solium* which infect the human brain, a site impervious to the immune response, are protected from destruction by the host. Similarly, plerocercoids of the fish tapeworm, *Proteocephalus ambloplitis*, avoid shedding from the intestine of its host by migrating into parenteral sites as the water temperature declines in winter, a period when feeding is reduced and worms voided because of a lack of nutrients, [10]. However, the parasites reenter the intestine as the water temperature increases in spring to mature as adult tapeworms. Some tissue-invading parasites can become encapsulated by fibrous tissue in their hosts as a result of the latter's defence mechanism and appear as cysts. They might remain viable for lengthy periods until acquired by new and/or appropriate definitive hosts. Xenomas of microsporans such as *Loma branchialis* in salmonids, species of *Sarcocystis* in birds and mammals, and cysticercoids of tapeworms as *Taenia saginata* in cattle and *Trichinella spiralis* in pigs use this strategy to infect new hosts following ingestion. Encapsulation can also be viewed as a defence mechanism by the host to curtail tissue damage by some migrating parasites. Mathematical models have been proposed to explain some host-parasite interactions [11]. However, Sures [12] discussed host-parasite interactions based on observations that some intestinal fish parasites accumulate heavy metals and can be useful as diagnostic tools to determine bioavailability. The approach, in this presentation will focus on some factors that influence fish-parasite interactions including disease aspects, climatic change, and environmental pollution.

 The immune response to foreign proteins in fish is lower in magnitude compared to mammals. Some of the defense barriers include mucous in the skin and gills, bile, digestive enzymes, and immunological barriers, primarily cellular and antibody responses. Some of these hinge on age of the host and ambient water temperature. A parasite's specificity will also determine if an infection becomes established. For example, a hemoflagellate, *Cryptobia salmositica*, is infective to some salmonid species but *Salvelinus namaycush* exhibits innate resistance [13]. Lytic antibodies were responsible for resistance but these apparently were absent in *S. fontinalis* that was susceptible to the infection. Cellular response is observed when some tissue-invading parasites become encapsulated. The immune response is temperature dependent as larval anisakine nematodes ingested in late autumn-winter, when water temperatures range from 0 to 4 EC, remain free in the tissues of Atlantic cod, *Gadus morhua*, whereas in summer, most become encapsulated (Khan, unpubl. data).

 Some environmental factors have a profound effect on several fish-parasite interactions. Generally, ectoparasites differ from endoparasites as the defence mechanisms tend to be reduced externally in fish. The interaction between an ectoparasitic ciliate, *Trichodina murmanica*, and its host, the Atlantic cod, is age and temperature-dependent. Prevalence and abundance of the parasite on the skin of 1-year juvenile fish in nature are extremely low and rare in older fish. However, outbreaks have occurred in fingerling and 1-year fish cultured cod held in over-stocked conditions during winter when water temperature was 0-1°C [14]. The infection and mortality declined with increasing water temperature and were rarely seen during summer at 8–14°C. Infection of Atlantic cod with *Trypanosoma murmanensis* was also age and temperature dependant as mortality was greater in younger than older fish [15]. Moreover, the infection persisted for longer periods (6–8 weeks) at lower (0–2°C) than higher (10–12°C) temperature (Khan, unpubl. data). It is probable that the host's immunity is temperature-dependent or the parasite is adapted to low temperatures as noted in some subarctic marine leeches [16]. In contrast, a microsporan, *Loma branchialis*, appearing as macroscopic cysts on the gills of fingerling and juvenile cod, caused die-offs only in summer-autumn when water temperatures were high [17]. Die-offs have also occurred in commercial-size cod held in sea pens for market in summer (Barker, unpubl. data). Xenomas resembling tumours, occurred in all the internal organs and moribund fish, in an emaciated condition, succumbed in a matter of weeks. Similar temperature-related die-offs have occurred in cultured juvenile Arctic charr, *Salvelinus alpinus*, only in summer following infection with a myxozoan, *Tetracapsuloides bryosalmonae*, held in earthen ponds [18]. Outbreaks of disease caused by plerocercoids of a cestode, *Diphyllobothrium dendriticum*, occurred in cultured rainbow trout, *S. namaycush*, after transfer from a hatchery to an embayment only in summer [18]. Host response to the parasites was minimal except for *Loma branchialis* as encapsulation of the xenomas did occur but some ruptured releasing spores that presumably infected other organs and tissues. Influence of water temperature on host-parasite interactions has also been reported previously [19].

 Some parasitic crustaceans interacting with their fish hosts can have a profound impact on their health [20]. The pennellid copepod, *Lernaeocera branchialis*, anchors its holdfast into one of the branchial blood vessels causing anemia and mortality depending on the age of the fish and the number of infecting parasites [21]. Mortality was high in juvenile cod about 3 years old but declined with increasing age [21]. Additionally, fish with multiple numbers of parasites were also likely to succumb. However, some fish that shed their parasites previously exhibited complete recovery. Prevalence and abundance of the infection varied with location in coastal Newfoundland [22]. Commercial-size Atlantic cod that were held in sea cages during the summer also succumbed when water temperatures were high but declined during winter [23]. Smith et al. [24] reported that the parasite induced extensive hyperplasia in the gills, intravascular thrombus formation and moderate cellular response in the cardiac and branchial tissues. In contrast, hundreds of larval stages that attach to the gills of the intermediate host, the lumpfish, *Cyclopterus lumpus*, cause no effect [22].

 Another gadid fish, the rock cod, *Gadus ogac*, that inhabits inshore embayments off St. Lewis, Labrador (52°22′N, 55°41′W), appeared to tolerate multiple numbers of parasites without exhibiting debility in contrast to Atlantic cod. (Khan, unpubl. data). Young fish were infected with a fewer mean number of parasites than older cod, some with as many as 7 parasites per older host (Table 1). Prevalence of the infection was 84% in 1976 and mean abundance increased with the length of the fish. Although fewer samples were caught in 1986 (as a result of a population decline triggered by climatic changes), there was a slight increase in prevalence but no significant change in mean abundance. Unlike Atlantic cod, the infected rock cod appeared robust and the gills pink to red without any indication of an anaemic condition observed in other gadids [21]. Prevalence of the infection was considerably lower in Atlantic cod captured inshore by cod trap in both 1976 (4% of 48 fish) and in 1986 (5% of 36 fish). The rock cod is cold-water-adapted fish that lives inshore beneath the ice in winter and apparently was not affected by oceanographic changes that occurred from the mid-1980s off eastern Canada [25].

 Marine hematophagous leeches (Hirudinea: Piscicolidae) exhibit an interesting interrelationship with their fish hosts. Some species of leeches feed on a variety of teleosts while others tend to be host specific. *Johanssonia arctica*, a deep-sea species adapted to subarctic conditions, fed on several species of teleosts while two species of *Malmiana, M. scorpii* and *M. brunnea*, were found only on shorthorn (*Myoxocephalus scorpii*) and longhorn sculpins (*M. octodecemspinosus*), respectively, in the NW Atlantic Ocean [26, 27]. Several species of marine leeches attach to the skin of their hosts to feed on blood but others, such as *Oxytonstoma microstoma* and *O. sexoculata*, adhere to the gills and on the angles of the oral cavity, respectively [26, 27]. These previously mentioned leeches remain permanently attached to their hosts, feeding intermittently until maturity, copulation, and cocoon deposition [26, 27]. Others, such as J. *arctica*, *Myzobdella lugubris,* and *Notostomum cyclostomum*, after feeding on fish, detach and reattach to crabs (Crustacea: Decapoda) for transport and deposition of cocoons [9, 16, 28, 29]. Locating fish hosts for a blood meal is increased by the foraging activities of the crabs. Other leeches deposit their cocoons on the egg masses of their piscine hosts or on rocks frequented by fish [16]. Synchronous hatching of larval fish and young leeches ensures that the latter can locate a host for their subsequent blood meals. The quantity of blood extracted by leeches varies considerably depending on size and species [9]. Mace and Davis [30] reported slow growth rate and energy loss in shorthorn sculpins infected with *M. scorpii*. Hematophagous leeches can induce anemia, subcutaneous hemorrhage, and inflammation especially when a heavy infestation occurs [31]. Moreover, leeches can also transmit blood parasites during hematophagy. *J. arctica* is capable of transmitting a trypanosome, *T. murmanensis*, a piroplasm, *Haemohormidium beckeri*, and probably a hemogregarine, *Haemogregarina uncinata* [32–34]. Another genus of hemoflagellates, species of *Trypanoplasma* (*Cryptobia*) is transmitted by leeches [35]. *Trypanoplasma bullocki* has caused mortality in summer flounder, *Paralichthys dentatus*, populations in the Middle Atlantic Bight [36]. Consequently, interaction between hematophagous leeches and fish can result in stress as a result of blood loss and also in the transmission of pathogenic parasites [32].

 Some parasites are known to predispose their hosts for predation by alteration of their behavior as in some carnivore-herbivore interactions. In the three-spine stickleback, *Gasterosteus aculeatus*, a larval cestode, the plerocercoid of *Schistocephalus solidus*, infects the body cavity and can impair swimming. It has been reported that infected fish are more likely to be predated than uninfected sticklebacks [37, 38]. Some fish with distended abdomens have been observed swimming near the surface in ponds in Newfoundland (Khan, unpubl. data). Examination of the fish with swollen abdomens revealed at least two large larvae per host. It is likely that sea gulls, *Larus* spp., that frequented these areas, were feeding on the fish. The Arctic tern (*Sterna paradisaea)* is also a definitive host of *S. solidus*. Nestlings on a small island near Cow Head (49°55′N, 57°53′W), Newfoundland, were fed sticklebacks by the parental birds, and during one wet and cool summer, several nestlings were observed in an emaciated condition. Predation of nestlings by sea gulls was apparent in the area. Examination of nine freshly dead birds revealed cestode larvae in the coelomic cavity of six carcasses ($\overset{-}{x}\;\;3.9\pm 1.2$/nestling), all exhibiting evidence of hemorrhage in the body cavity and an absence of food in the digestive tract (Khan, unpubl. data). It is likely that the parasite, lacking nutrients in the digestive tract, migrated from this site through the coelomic wall, causing the lesions observed and also predisposed them to predation by foraging gulls.

 An unusual difference in the abundance and prevalence of parasites was observed in two populations of landlocked Arctic charr inhabiting different habitats in a pristine deep-water lake in Gander (48°58′N, 54°57′W), Newfoundland (Khan, unpubl. data). One of these, a pale-colored morph, pelagic and living in shallower water, fed primarily on mayfly nymphs, *Heptagenia* spp. (Ephemeroptera: Heptageniidae) and other insect larvae were more parasitized than the dark morph inhabiting a mid-water-benthic zone feeding on macroinvertebrates and fish such as sticklebacks. DNA evidence has revealed that the two populations were distinct and might have been separated a long time previously probably during the postglacial period [39]. Meristic results and colouration have revealed that mixing was rare, with each group occupying different niches. Species diversity, abundance, and prevalence of the parasitic helminth taxa, trematodes, cestodes, and nematodes were significantly greater in the pelagic than in the mid-water-benthic group (Table 2). These results are reminiscent of a hybrid salmonid, the splake (*Salvelinus fontinalis* ×* S. namaycush*), a cross between a brook trout (*S. fontinalis*) and lake trout (*S. namaycush*), that was bred to avoid lamprey (*Petromyzon marinus*) predation by inhabiting mid-water rather that the benthic area where lake trout frequented [40]. Fewer parasitic species (23 spp.) were observed in the splake than in the lake trout (75 spp.) [41]. The Arctic charr is a host to several species of metazoan parasites [41]. Factors responsible for the separation of the two Arctic charr populations in Gander Lake remain enigmatic but they suggest an example of parasite paucity resulting from habitat selection.

 Oceanographic changes caused by a series of adverse climatic events have also had an impact on host-parasite interactions especially on the abundance and prevalence of metazoan parasites in the digestive tract of Atlantic cod occurring off the coast of Labrador, Canada [42]. Changes in the climate caused water temperatures to decrease resulting in a decline of ocean fish and sea birds [25]. Prior to this time, the abundance of an acanthocephalan parasite, *E. gadi,* was high but, following a chain of cascading events during the mid-1980s, it decreased to extremely low levels [43]. Outmigration of the main food source, the capelin, *Mallotus villosus*, and also the paratenic host of the infection in older Atlantic cod, was the underlying cause [43]. Decline of the abundance of *E. gadi* should favour its fish host as the spines on the proboscis of some acanthocephalans are known to cause lesions in the intestinal wall and ultimately affect growth [44]. However, the abundance of cod in the area continues to be low as a result of a sparcity of capelin [45].

Fish parasites can also be useful as bioindicators of habitat degradation caused by anthropogenic contaminants especially when sensitive species are sampled as sentinels. These bioindicators include abundance, prevalence, and species diversity. These variables might increase or decrease following long-term exposure. Effluent, discharged by two pulp and paper mills in Newfoundland, caused both external and internal lesions, disrupted gonadal development, and altered length-class distribution in all age groups of winter flounder, a sediment-inhabiting flatfish species [46, 47]. In both inlets, the fish were infected with large numbers of metacercaria of a digenetic trematode, *Cryptocotyle lingua*, on the body, head, and fins compared to reference samples. Low levels of lymphocytes in the heavily parasitised fish were most likely indicative of a compromised immune system [48]. Abundance of *C. lingua* was also high in nursery areas of the flounder where untreated municipal effluent, containing sewage- and crank-case petroleum waste, was discharged (Khan, unpubl. data). It appears that an abundance of food for fish and sea gulls (*Larus* spp.), definitive hosts of the parasite, attracted them to these areas. Additionally, macroscopic xenomas of another parasite, a microsporan, *Glugea stephani*, occurred in the internal organs including the heart, liver, spleen, kidneys, intestine, and gonads in samples taken near the mill whereas they were restricted to the wall of the digestive tract of reference samples [49]. Lower than normal lymphocyte levels associated with host resistance probably provided an opportunity for the parasite to metastasise following release of spores from ruptured xenomas to infect other sites. Another study reported reduced numbers of digeneans and myxozoans but increased numbers of acanthocephalans in roach (*Rutilus rutilus*) and perch (*Perca fluviatilis*) in a lake receiving effluent from a pulp mill when compared to samples from two less polluted oligotrophic lakes [50]. Changes in the density of the intermediate hosts, toxic effect on the ectoparasites, and impairment of the immune response were suggested as the underlying causes.

Both field and laboratory studies have revealed that some parasites of winter flounder respond differently at various concentrations to discharges from a pulp and paper mill [47, 51, 52]. Gradient sampling of winter flounder inhabiting a fjord where pulp and paper mill effluent had been discharged for several decades revealed that two selected helminths, a digenean, *S. furciger*, and an acanthocephalan, *E. gadi,* increased in abundance down current from the outfall [47]. External and internal lesions, low body condition, and organosomatic indices, but elevated levels of detoxifying enzymes in the liver, were also noted in the affected fish [53]. Winter flounder captured from a pristine site were exposed to sediment collected at the four sites down current from the discharge. The results provided evidence to support the field study that enteric parasites were more abundant in flounder taken from the farthest location than others originating from the proximity of the paper mill [52]. Histopathological changes confirmed that the fish were exposed to toxic chemicals. These results suggest that host-parasite interactions can be affected after chronic exposure to anthropogenic discharges.

 The balance between host and parasite interactions was also affected in sculpins and winter flounder living in coastal habitats where untreated domestic sewage and polychlorinated biphenyls (PCBs) were disposed. Untreated domestic sewage was responsible for an increase in the abundance of *Trichodina* spp. and *Gyrodactylus pleuronecti* on the secondary gill lamellae of shorthorn sculpins sampled in an embayment located in eastern Newfoundland. The trichodinids were more abundant ($\overset{-}{x}$, 3.6 ± 0.4; *n* = 16) where the sewage was discharged than at one ($\overset{-}{x}$, 4.1 ± 1.3; *n* = 14) or 5 km ($\overset{-}{x}$, 2 ± 0.3, *n* = 23) offshore (Khan unpubl. data). Khan and Hooper [54] noted that abundance and prevalence of ectoparasitic ciliates and enteric helminths increased with distance from the point of discharge of thermal effluent in winter flounder. However, myxozoans in the gall bladder were more prevalent at the discharge location than down current. Sculpins sampled at a site where PCBs had been discharged exhibited also a greater abundance of trichodinids on the gills and myxozoans in the gall bladder of the sculpin, *M. *scorpius, than at the reference site, while enteric helminthes and ectoparasitic leeches were fewer than in latter fish [55]. Moreover, 14 species of parasites occurred in the PCB-affected sculpins in contrast to 11 in the reference samples [55]. External and toxicopathic lesions in several tissues, significantly lower body condition and organ somatic indices as well as lower hemoglobin and lymphocyte levels, were noted in the PCB-contaminated fish when compared to reference fish. Lack of parasite diversity was likely associated with the brackish water conditions that affected the transmission of some sensitive parasite species in the reference samples.

Studies on winter flounder exposed to petroleum aromatic hydrocarbons (PAHs), both in the field and in the laboratory in a dose-response trial, revealed a similar result [56]. Ectoparasites such as trichodinid ciliates and a monogenean increased to a peak but declined as the concentration increased while enteric helminthes declined progressively. Similar results were observed when species of sculpins (*Myoxocephalus* spp.) were exposed to PAHs. Trichodinid ciliates and monogeneans infecting the gills of sculpins and Atlantic cod, respectively, also increased in abundance or prevalence following chronic exposure to PAHs ([57, 58] and references therein). Additionally, exposure to PAHs and a concurrent infection with the hemoflagellate, *T. *murmanensis, caused greater mortality and a greater abundance of parasites in both Atlantic cod and winter flounder than in fish infected only with the parasite or exposed only to PAHs [59]. In contrast, endoparasitic helminthes declined after exposure, with the PAHs probably simulating antihelminthic drug action and/or changes in host physiology [56, 60]. However, ectoparasitic abundance hinged on the concentration of the pollutant as it declined after a peak with increasing levels of the PAHs [56]. Fewer species and their abundance of both groups of parasites were also observed in sculpins exposed to PCBs at an impacted military dockyard than at a reference site as noted previously [55]. Increase of ectoparasites was probably associated with a decline of the host's immune response, hyperplasia in the secondary gill lamellae, excessive epidermal mucus secretion, and exfoliation that attracted opportunistic bacteria which served as additional food for them. Changes in host physiology, toxicity of the contaminants to the larval and adult stages of the helminths as well as to their intermediate hosts, especially in the field, might have been responsible for their decline. Sanchez-Ramirez et al. [61] also reported that a monogenean *Cichlidogyrus sclerosus* increased in abundance after exposure of Nile tilapia (*Oreochromis niloticus*) to sediment contaminated with PAHs, PCBs, and heavy metals in a static system for 15 days. The sediment induced immunosuppression and caused histological anomalies in the gills and spleen. Future studies on fish parasites as bioindicators should include additional information on the fish's body condition, organ somatic indices, histopathological effects, and also hepatic detoxifying enzymes [51].

 Acid precipitation has also affected host-parasite interactions in fish. Parasite richness in eels (*Anguilla rostrata*), including monogeneans and digeneans, was greater in less acidified locations than in more acidic sites in Nova Scotia [62]. Other parasites including acanthocephalans and copepods did not appear to be affected.

 In summary, observations on host-parasite interactions are complex, at times difficult to interpret on account of a number of variables that can shift the balance one way or the other. Factors such as host's age, behaviour, immunological competence, and environmental change can play a role in the association. Alternatively, establishment and evasion by the parasite of the host's responses appear to be significant factors. Consequently, the outcome in this interaction will hinge on host susceptibility and resistance and the parasite's ability to infect its host. It is suggested that future studies, investigating host-parasite interactions in habitats degraded by anthropogenic contaminants, should consider sampling multiple sites, especially along a gradient, and include more than one bioindicator and sensitive fish species. 

## Figures and Tables

**Table 1:** Abundance ($\bar{x}$  ± s.e.) and prevalence (%) of the copepod, *Lernaeocera branchialis*, on the gills of the rock cod, *Gadus ogac*, sampled in Labrador in 1976 and 1986.

Year	Fish length (cm class)	No. infected/ no. examined	$\bar{x}$ ± s.e.	%
1976	21–30	9/17	1.8 ± 0.2	53
	31–40	6/12	2.1 ± 0.2	50
	41–50	21/21	3.3 ± 0.4	100
	51–60	24/24	3.1 ± 0.3	100

1986	31–40	2/3	3.5 ± 0.5	67
	41–50	4/4	3.1 ± 0.4	100
	51–60	3/3	3.0 ± 0.4	100

**Table 2:** Comparison of the abundance ($\bar{x}$  ± s.e.) and prevalence (%) of some metazoan parasites found in the digestive tract of two populations of Arctic charr (*n* = 25) living near the surface and in the midwater benthic zone of Gander Lake, Newfoundland.

Parasite taxa	Parasite species	Fish groups			
		Surface		Midwater/benthic	
		$\bar{x}$ ± s.e.	%	$\bar{x}$ ± s.e.	%
Trematoda	*Bunodera lucioperca*	<0.1	12	—	—
	*Crepidostomum* sp.	<0.1	8	—	—
Cestoidea	*Eubothrium salvalini*	3.2 ± 0.4	100	<0.1	12
	*Proteocephalus* sp.	<0.1	32	—	—
Nematoda	*Pseudocapillaria salvelini*	2.1 ± 0.2	100	<0.1	8
Acanthocephala	*Echinorhynchus lateralis*	<0.1	20	—	—
